# Extraction, purification, structure, modification, and biological activity of traditional Chinese medicine polysaccharides: A review

**DOI:** 10.3389/fnut.2022.1005181

**Published:** 2022-09-09

**Authors:** Hongkun Xue, Pengcheng Li, Jiayue Bian, Yuchao Gao, Yumei Sang, Jiaqi Tan

**Affiliations:** ^1^College of Traditional Chinese Medicine, Hebei University, Baoding, China; ^2^College of Food Science and Technology, Jilin Agricultural University, Changchun, China; ^3^School of Basic Medical Sciences, Hebei University, Baoding, China; ^4^Medical Comprehensive Experimental Center, Hebei University, Baoding, China

**Keywords:** traditional Chinese medicines, polysaccharides, extraction and purification, structure, biological activities

## Abstract

Traditional Chinese medicines (TCM), as the unique natural resource, are rich in polysaccharides, polyphenols, proteins, amino acid, fats, vitamins, and other components. Hence, TCM have high medical and nutritional values. Polysaccharides are one of the most important active components in TCM. Growing reports have indicated that TCM polysaccharides (TCMPs) have various biological activities, such as antioxidant, anti-aging, immunomodulatory, hypoglycemic, hypolipidemic, anti-tumor, anti-inflammatory, and other activities. Hence, the research progresses and future prospects of TCMPs must be systematically reviewed to promote their better understanding. The aim of this review is to provide comprehensive and systematic recombinant information on the extraction, purification, structure, chemical modification, biological activities, and potential mechanism of TCMPs to support their therapeutic effects and health functions. The findings provide new valuable insights and theoretical basis for future research and development of TCMPs.

## Introduction

Traditional Chinese medicine (TCM) is one of the most important parts of Chinese culture and medical practice. In recent decades, increasing researches have confirmed that the leaves, stems, flowers, fruits, and tubers of TCM contain a variety of bioactive compounds such as polysaccharides, polyphenols, proteins, amino acid, fats, vitamins, and other components (1, 2). TCM polysaccharides (TCMPs), as a kind of biological macromolecules, are composed of 10 or more monosaccharides (3), and TCMPs with different biological activities have become the latest research hotspot. Each monosaccharide molecule is connected by glycosidic bonds and can be represented by the general formula (C_6_H_10_O_5_)n (4). According to statistics, at least 30 kinds of TCMPs have been investigated in standard clinical trials. Growing studies have indicated that TCMPs show various biological activities, such as antioxidant, anti-aging, immunomodulatory, hypoglycemic, hypolipidemic, anti-tumor, anti-inflammatory, and other activities (5, 6). In addition, chemical modifications can improve or change the biological activities of TCMPs and have attracted extensive attention (7). Furthermore, TCMPs show little toxic and side effects. Thus, TCMPs, as a natural active components, have been extensively used in cosmetic, food, and health product industries.

**Figure 1:**
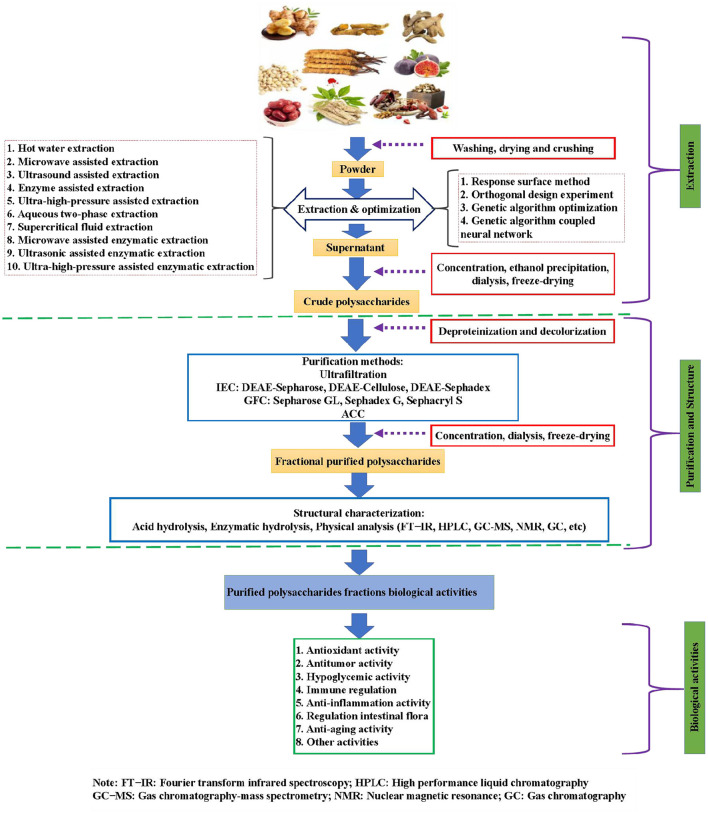
The schematic diagram of extraction and purification methods, structural characterization, chemical modification, and biological activities of TCMPs.

At present, the researches on TCMPs mainly focus on theirs extraction, purification, structural characterization and modification, and biological activities (6). Nowadays, there are various extraction methods of TCMPs, which often affect the types, physicochemical properties, and biological activities of the final polysaccharides extraction products (8). Currently, hot water extraction (HWE) is the main method of polysaccharides extraction because it has the advantages of convenient extraction, no special equipment, and low cost (9). However, some polysaccharides are insoluble in hot water, and this technology can not realize the effective extraction of polysaccharides. Hence, some new extraction methods and techniques have been proposed for the extraction of polysaccharides with different properties (Figure 1). Nevertheless, the purity of polysaccharide is still low due to the limitation of extraction technology. Hence, the purification and structural identification of TCMPs are particularly important. As we all know, the purification of TCMPs is very complex, and it is difficult to obtain high-purity homogeneous polysaccharides fractions (10). Increasing studies have confirmed that the extraction and purification methods have an important impact on the modification and biological activities of polysaccharides (11–13). Consequently, the extraction and purification of TCMPs are an important prerequisite for the analysis of their biological activities and chemical modification. TCMPs can be used as natural candidate drugs for the prevention and treatment of different diseases based on the existing literature. Thus, the research progress and future prospects of TCMPs must be systematically reviewed to better understand TCMPs. In this review, the studies on the extraction and purification methods, structure, chemical modification, and biological activities of TCMPs in recent years were summarized (Figure 1), and the research progress and future development trend were described in detail. This review provides an important scientific basis for further in-depth development and utilization of TCMPs.

## Extraction and purification of TCMPs

With the progress of science and technology, the extraction and purification technologies of polysaccharides have been fully developed. The nutritional and medicinal values of TCMPs have attracted extensive attention. The various traditional and novel extraction methods have already been established and proposed (6). Table 1 summarizes the different extraction, purification methods, and the corresponding TCMPs yield. According to the distribution and cell localization of TCMPs, and TCMPs are mainly divided into intracellular and extracellular polysaccharides (54). It is worth noting that the crude extracellular polysaccharides can be directly extracted by ethanol precipitation method (55). As for intracellular TCMPs, they are mainly extracted from the leaves, stems, flowers, fruits, and tubers of TCM by using different extraction methods (56–58). Before extracting intracellular TCMPs, the raw materials need to undergo a series of pretreatment, including washing, drying, crushing, and degreasing. Currently, hot water extraction (HWE) is the most commonly used and convenient extraction method, which is usually used to extract polysaccharides from natural resources in laboratory and industrial applications (59–61). Additionally, dilute alkali/acid methods, as another traditional extraction method, can improve the extraction efficiency and reduce the separation time of TCMPs to a certain extent (62). Nevertheless, the acidic and alkaline conditions must be strictly controlled to avoid the structure destruction of TCMPs. All in all, the traditional extraction methods have some advantages. However, they have significant disadvantages, such as low efficiency, long extraction time, large solvent consumption, and high extraction temperature (6). Hence, these methods are not suitable for large-scale extraction of polysaccharides in industry.

**Table 1:** Extraction, purification, structural characterization, biological activities of polysaccharides from traditional Chinese medicines.

**Sources**	**Extraction methods**	**Yield**	**Purification** **methods**	**Compound** **name**	**M_W_/kD**	**Monosaccharide composition(mol% or mole ratio)**	**Analysis** **technique**	**Structure**	**Biological** **activities**	**References**
Black mulberry fruit	HWE	11.86% ± 0.21%	DEAE-52, Sephadex G-100	BMP-1-1	615	Man, Rha, GlcA, Gal, Ara	FT–IR, HPGPC	NO	Antioxidant	(14)
				BMP-2-1	405	Rha, GlcA, GalA, Gal, Ara				
*Turmeric*	HWE	2.23 g/100 g	Anion-exchange chromatography	TPs-0	ND	Rha, Glu, Gal, Ara, Xyl, galacturonic acid, and glucuronic acid	GC, GC-MS, NMR	α-Araf- (1 → 4) -α-Glcp- (1 → 3) -α-Arap- (1 → 3) -β-Galp- (1 → 3,6) -α-Galp- (1 → 5) -α-Araf- (1 → 3) -β-Galp- (1 → R	Antioxidant	(15)
				TPs-1						
				TPs-2						
				TPs-3						
*Laminaria japonica*	HWE	13.31% ± 0.08%	DEAE glucan gel A-25	LGPs	17.12	Rha, Fuc, Xyl, Man Glu, Gal = 4.51:20.27:12.43: 12.81:10.29: 39.69	UV, FT–IR, GC, HPGPC, NMR	NO	Antioxidant	(16)
*Ganoderma lucidum*	HWE	ND	Sephadex G-50	ASP	80	GlcA:Glc:Ara:Gal = 1.00:1.70:1.85:5.02	UV, FT–IR, HPGPC	NO	Anti-tumor	(17)
Longan fruit Pericarp	HWE	ND	Sephadex G-100	PLFP	420	Ara:Glc:Gal:GalA = 32.8:17.6:33.7:15	HPGPC, GC, GC–MS, FT–IR, NMR, Methylation	→ 5)-l-Araf-(1 →, → 6)-d-Glcp-(1 →		(18)
Longan pulp	HWE	ND	IEC, GFC	LPIIa	44.7	ND	HPGPC, GC, GC–MS, FT–IR, NMR, Methylation	→ 6)-Glc-(1 →, → 5)-Ara-(1 →, → 4)-Man-(1 → and → 6)-Gal-(1 →.	Immunomodulatory	(19)
*Schisandra chinensis*	HWE	ND	Anion-exchange chromatography	SSP, SCP	ND	Ara, Glc, Gal	HPGPC, FT–IR	ND	Antioxidant, Immunomodulatory	(20)
Mulberry	UAE	3.13%	Anion-exchange chromatography	MFP	ND	ND	FT-IR	ND	Antioxidant, hyperglycemic	(21)
Longan fruit pericarp	UAE	15.41%	ND	PLFP	ND	ND	ND	ND	ND	(22)
*Glycyrrhiza*	UAE	3.53%	ND	GP	ND	ND	ND	ND	Antioxidant	(23)
*Taraxacum officinale*	UAE	6.46% ± 0.19%	DEAE-52, Sephadex G-100	Neutral polysaccharide	1.7	ND	FT–IR, HPGPC, Methylation analysis, NMR	(1 → 4)-β-D-Glcp with branching at O-2 of (1 → 2,4)-β-D-Glcp	Antioxidant, anti-tumor	(24)
*Radix polygonati officinalis*	UAE	ND	DEAE-52, Sephadex G-100	LLPS-1	350.5	Glc:Man = 1:2	FT–IR, HPGPC	NO	Immunomodulatory	(25)
				LLPS-2	403.3	Glc:Man = 1:1				
				LLPS-3	146.2	Ara:Gal:Glc:Man = 2:2:2:1				
*Chrysanthemum morifolium* cv.	UAE	8.29% ± 0.18%	ND	ND	ND	ND	FT–IR	NO	Antioxidant, α-glucosidase activities	(26)
*Tangerine* peels	MAE	19.9% ± 0.2%	AEC, GFC	TPPs-2-1	17.8	GalA, Ara, Gal, Rha, Glc, Man	FT–IR, HPGPC, GC–MS	NO	Antioxidant	(27)
*Eucommia ulmoides* Oliver leaf	MAE	12.31%	Anion exchange chromatography	EULP	38.83	Rha, Ara, Gal, Glu, Xyl, Man, glucuronic acid, and galacturonic acid = 7:4:6:14:1:2:3:1	GC–MS, GC, FT–IR, HPGPC	NO	Antioxidant	(28)
*Salvia miltiorrhiza* Bunge	MAE	14.11%	DEAE-52, Sephadex G-100	SMP1	6.087	Glu (1%), Gal (1.67%), and Fru (1.12%)	FT–IR, HPGPC, NMR	NO	Antioxidant	(29)
*Fructus Meliae Toosendan*	MAE	15.75%	Anion exchange chromatography	FMTP	1.288	NO	UV, FT–IR, HPGPC	NO	Antioxidant	(30)
Mulberry	EAA	ND	Anion exchange chromatography	MOS-1a	0.987	NO	UV, FT–IR, GC–MS, GPC	NO	NO	(31)
*Cornus officinalis*	EAE	9.29% ± 0.31%	Anion exchange chromatography	COP	NO	NO	NO	NO	NO	(32)
*Astragalus membranaceus*	EAE	29.96% ± 0.14%	ND	ND	ND	ND	ND	NO	Antioxidant	(33)
Korean ginseng	EAE	ND	Anion exchange chromatography	FGWP		Rha, Ara, Gal galacturonic acid, Glu = 1.8:10.1:9.2:17.8:60.6	UV, FT–IR, GC–MS, HPGPC	ND	Immunomodulatory	(34)
Alfalfa	EAE	5.05% ± 0.02%	ND	ND	ND	ND	ND	ND	Antioxidant	(35)
*Phellinus linteus*	ATPE	63.58% ± 1.12%	Anion exchange chromatography	C-PLPS	15.2	Ara, Xyl, Man, Glc, and Gal with the molar ratios of 1.0:1.5:3.4:25.2:1.1	UV, FT–IR, HPGPC	ND	Antioxidant, antitumor	(36)
*Lycium barbarum* L	ATPE	24.79 mg/g	DEAE-52, Sephadex G-100	PTP-1	ND	ND	FT–IR, UV	NO	Antioxidant	(37)
				PTP-2						
				PTP-3						
*Grifola frondosa*	ATPE	90.21%	Anion exchange chromatography	CGFP	2,954	Glu	FT–IR, AFM, HPGPC	NO	Antioxidant	(38)
*Schisandra chinensis*	ATPE	0.95 mg/g	NO	NO	NO	NO	NO	NO	NO	(39)
Cornus officinalis fruit	UATPE	7.85% ± 0.09%	DEAE-52 and Sephadex G-100	COPs-4-SG	33.64	Galacturonic acid, Ara, Man, Glu, and Gala in a molar ratio of 34.82:14.19:6.75:13.48:12.26.	FT–IR, AFM, CD, HPGPC	NO	NO	(40)
*Ziziphus Jujuba* cv.	UATPE	8.18%	Anion exchange chromatography	ZMP	NO	Rha, Ara, Xyl, Ma, Glu, Gal, and galacturonic acid with at the ratios of 1.46:2.47:2.27:1.12:1.00:1.57:5.40	HPGPC, UV, FT–IR	NO	Antioxidant	(41)
*Lilium davidiivar*. unicolor salisb	UATPE	36.58%	DEAE-52, Sephadex G-100	LPS	NO	NO	UV, FT–IR	NO	Antiglycation activity	(42)
*Lilium lancifolium* thunb	UATPE	15.17% ± 0.21%	DEAE-52, Sephadex G-100	LLPs-2-SG	421.41	Man, Glu, and Gal in a molar ratio of 10.52:23.06:7.19	UV, FT–IR, HPGPC, AFM, SEM	NO	NO	(43)
*Lonicerae japonica* leaves	UAEE	14.76%	DEAE-52, Sephadex G-100	LJLP	NO	Gal (32.3%), Glu (20.9%), and Rib (15.2%)	UV–vis, FT–IR, HPGPC	NO	Antioxidant	(44)
*Momordica charabtia* L.	UAEE	29.75% ± 0.48%	Anion exchange chromatography	MCP	NO	NO	NO	NO	NO	(45)
*Cucurbita moschata*	UAEE	4.33% ± 0.15%	Anion exchange chromatography	CMCP	NO	NO	NO	NO	Antioxidant	(46)
Corn silk	UAEE	4.56%	Anion exchange chromatography	CSPS	105.2	Rha, Ara, Xyl, Man, Gal = 4.17:17.33:5.59:18.65:19.11	UV, FT–IR, HPGPC	NO	Antioxidant and anticancer activities	(47)
*Armillaria* mellea	UAEE	40.56%	NO	NO	NO	NO	NO	NO	NO	(48)
*Hylocereus* undatus	UAEE	16.64%	NO	NO	NO	NO	NO	NO	NO	(49)
*Ocimum album seed*	HWE	7.1%	DEAE-52 and Sephadex G-200	OAP-1A	593	Man (35.7%), Glu (33.32%), Gal (19.6%), and Rha (11.38%)	GC–MS, NMR, FT–IR, HPGPC	→ 3)-β-D-Manp-(1 →, → 3,4)-β-D-Manp-(1 →, → 3,6)-β-D-Manp-(1 →, → 3)-α-D-Glcp-(1 → α-D-Glcp-(1 →	Antioxidant	(50)
*Glycyrrhiza inflata residue*	HWE	NO	DEAE-52 and Sephadex G-150	AGP	2.89	Rha: Ara:Xyl: Man: Glu: Gal with a molar ratio of 1:2.33:2.85:0.69:3.05:1.54	GC–MS, periodate oxidation, Smith degradation, FT–IR, methylation, NMR	→ 6)-β-d-Glcp-(→ backbone	α-glucosidase inhibition activity	(51)
*Laminaria japonica*	HWE	NO	diethylaminoethyl-cellulose and Sephacryl S-500 chromatography	LJP12	2.31	Ara:Xyl:Man:Glc:Gal = 1:0.17:1.54:2.64:0.18	HPGPC, FT–IR, NMR	1,4-linked and 1,3,6-linked	NO	(52)
*Laminaria japonica*	HWE	NO	DEAE-A25	WPS-2-1	80	Man:Rha:Fuc = 1.0:2.3:1.2	HPGPC, UV, FT–IR, NMR, Smith degradation	A backbone of array by (1 → 4)-glycosidic linkages	NO	(53)

HWE, Hot water extraction; UAE, Ultrasound assisted extraction; MAE, Microwave assisted extraction; EAE, Enzyme assisted extraction; UHPAE, Ultra-high pressure assisted extraction; ATPE, Aqueous two-phase extraction; UATPE, Ultrasound assisted aqueous two-phase extraction; UAEE, Ultrasound assisted enzyme extraction.

To solve the limitations of traditional extraction methods, some novel and effective extraction methods were proposed according to the principle of enhancing cell wall decomposition without destroying the structure of TCMPs. Notably, ultrasound assisted extraction (UAE), microwave assisted extraction (MAE), enzyme assisted extraction (EAE), and ultra-high pressure assisted extraction (UHPAE) methods have been widely used in the TCMPs extraction to improve theirs yield. Furthermore, the extraction conditions also affect the TCMPs yield to some extent. Thus, to improve the TCMPs yield, some optimization methods, including orthogonal experiments, response surface methodology (RSM), genetic algorithm (GA), and genetic algorithm coupled neural network (GA-NN) are used to optimize the extraction process of TCMPs. Wang et al. prepared polysaccharides from black mulberry by using HWE and optimized extraction process *via* RSM, and then purified crude polysaccharides by DEAE-52 cellulose and Sephadex G-100. The results show that the optimal extraction conditions were extraction time of 3 h, extraction temperature of 87 °C, and liquid-to-solid ratio of 39 mL/g. The yield of polysaccharides from black mulberry was 11.86% ± 0.21%. In addition, two purified fractions of BMP-1-1 with molecular weight of 615 kDa and BMP-2-1 with molecular weight of 405 kDa were obtained (14). Zhu et al. extracted the crude polysaccharides from turmeric (TPs) by HWE, optimized extraction conditions, and purified crude TPs by anion-exchange chromatography to obtain the four polysaccharides fractions, namely, TPs-0, TPs-1, TPs-2, and TPs-3, and then evaluated theirs antioxidant activity. The results show that the optimal extraction conditions are as follows: Extraction temperature of 100 °C, extraction time of 2.5 h, liquid-to-material ratio of 20 mL/g, and number of extractions of 2 times, and TPs yield was 2.23 g/100 g. TPs-0 and TPs-1 were mainly consisted of glucose (Glu), galactose (Gal), and arabinose (Ara), whereas TPs-2 and TPs-3 were composed of rhamnose (Rha), Glu, Gal, Ara, xylose (Xyl), galacturonic acid, and glucuronic acid. Moreover, TPs showed antioxidant effects on DPPH, ABTS^+^, and OH radicals, and the antioxidant effect of TPS-2 was the strongest, suggesting that TPs, as a natural antioxidant, are used in the development of functional foods and health products (15). Lu et al. prepared *Laminaria* japonica polysaccharides (LJP) with antioxidant capacity by using HWE and optimized extraction process *via* GA. The results show that the optimal process parameters were pH of 2.0, extraction temperature of 120°C, and extraction time of 3 h. The yield of LJP was 13.31% ± 0.08%. In addition, LJP showed good antioxidant activity including ORAC, ABTS^+^ radicals scavenging activity and reducing power, suggesting that LJP can be used as a natural antioxidant (16). Furthermore, HWE method is widely used to extract natural polysaccharides from *Ganoderma lucidum, Longan, Longan* pulp, and *Schisandra chinensis* (17–20). To sum up, HWE method shows some advantages. Nevertheless, the extraction efficiency of this method is low and TCMPs are easy to degrade due to the high extraction temperature. Thus, this method needs to be combined with other technologies to improve the extraction efficiency and expand its the application range.

UAE uses the “cavitation effect” and “mechanical shear effect” produced by ultrasound in the extraction process to destroy the plant cell wall, which can dramatically shorten extraction time, reduce energy and solvent consumption, and quickly achieve the purpose of extracting active ingredients (63). Thus, UAE has been widely used in the extraction polysaccharides from TCM. According to the full review of previous literature reports, TCMPs could usually be extracted by UAE under the following conditions: Extraction temperature of 57–80°C, liquid-to-solid ratio of 15–41 mL/g, ultrasound power of 120–600 W, and extraction time of 12 min−2 h (Table 1) (21–26). By analyzing the above literature reports, it was found that the yield of polysaccharides obtained by UAE was higher than that traditional HWE. A large number of studies have confirmed that higher ultrasonic frequency (above 100 kHz) could destroy the glycosidic bond and lead to the polysaccharides depolymerization, whereas the extraction efficiency of polysaccharides was not significant at low ultrasound frequency (20–50 kHz) (64, 65). Therefore, it is necessary to optimize the conditions of UAE.

MAE uses electromagnetic waves with the frequency of 300 MHz−300 GHz to selectively heat materials. The principle is to use microwave radiation to directly penetrate plant cells. Polar molecules absorb microwave energy, and the temperature in the cell rises rapidly, and then the solvent evaporates to produce cell pressure (66). The synergistic effect of concentration and pressure gradients promotes the diffusion of target components from the inside to the outside of the cell. In addition, the generated pressure can destroy the plant cell wall to enhance the extraction efficiency of active ingredients (67). Chen et al. extracted tangerines peel polysaccharides (TPPs) by using MAE and optimized the extraction process *via* RSM based on single factor experimental results. The results show that the optimal combination of process parameters was as follows: Microwave power of 704 W, extraction temperature of 52.2°C, and extraction time of 41.8 min, and the yield of polysaccharides was 19.9% ± 0.2% under the optimal extraction conditions (27). Xu et al. prepared *Eucommia ulmoides* Oliver leaf polysaccharides by MAE, optimized the extraction condition through RSM, purified the crude polysaccharides by different purification methods to obtain the homogeneous fraction (EULP) with the molecular weight of 38.83 kDa, and evaluated its antioxidant activity. It was found that the optimal process was obtained as follows: Microwave extraction of 74 °C, solid-to-liquid ratio of 1:29 g/mL, and extraction time of 15 min. The yield of polysaccharides was 12.31% under above extraction conditions. Additionally, the antioxidant activity of EULP was significantly better than the crude polysaccharides (28). Meng et al. extracted polysaccharides from *Salvia miltiorrhiza* Bunge (SMPs) *via* MAE, optimized the extraction process by RSM, purified the crude SMPs by using DEAE-52 and Sephadex G-100 chromatography to obtain a novel the homogeneous fraction (SMP1). The results show that the optimal process parameters are as follows: Microwave power of 1,200 W, liquid-to-solid ratio of 38 mL/g, extraction time of 12 min, and ethanol concentration of 86%. The yield of polysaccharides was 14.11% under above extraction conditions. SMP1 with an average molecular weight of 6.087 kDa was consisted of Glu (1%), Gal (1.67%), and Fru (1.12%). Pharmacological researches display that SMP1 protected from OGD/R-induced ferroptosis by activating Nrf2/HO-1 pathway in PC12 cells, suggesting that SMPs can inhibit iron sagging and reduce oxidative stress damage (29). Xu et al. prepared the polysaccharides from *Fructus Meliae Toosendan* (FMTP) by using MAE, optimized the extraction process *via* RSM based on single factor experimental results, purified the crude polysaccharides to obtain high-purity FMTP polysaccharide fraction with the molecular weight of 1.288 kDa. It was found that the optimum extraction conditions for FMTP: Solvent-to-liquid ratio of 30 mL/mg, extraction time of 20 min, extraction times of 2, and microwave power of 700 W, and the extraction rate of FMTP was 15.75% under the optimum extraction conditions. Furthermore, FMTP could effectively scavenge free radicals (DPPH, OH, and O^2−^), indicating that FMTP can be used as a potential antioxidant in functional foods and drugs. To sum up, MAE has the advantages of high extraction efficiency, energy conservation, and environmental protection. Nevertheless, microwave causes local high temperature in the extraction solution, resulting in polysaccharides degradation (30). Thus, this method needs to be combined with other extraction methods to expand the application range of microwave in the extraction field.

EAE uses biological enzymes as a biocatalyst to destroy the structure of plant cell wall, improves the permeability of cell membrane and cell wall, reduces the mass transfer resistance of active ingredients, and accelerates the dissolution of active components in cells, which can improve the yield of effective components (68). EAE has the advantages of mild reaction conditions, short extraction time, simple process, high efficiency, low investment cost and energy consumption. Moreover, the biological activities of target components have not been significantly reduced, and less chemical reagents are used in EAE, which is conducive to resource utilization and environmental improvement. Thus, EAE method has been used to extract natural polysaccharides from traditional Chinese medicines, such as mulberry, *Cornus officinalis, Astragalus membranaceus, Korean ginseng*, and *Schizochytrium limacinum* (31–35) (Table 1). However, the high price and strict extraction conditions of enzyme are difficult to control. Thus, the large-scale industrial application of this technology in the field of food and medical treatment is still very difficult.

Aqueous two-phase extraction (ATPE) refers to mixing two polymers or one polymer with an aqueous solution of a salt to form a two-phase aqueous system for the extraction active components (69). Its principle is to use the different partition coefficients of target components in two phases to extract and separate. This technology has mild conditions and wide biological adaptability. Therefore, it can be used in biological extraction, natural product separation and so on. Wu et al. prepared and separated the *Phellinus linteus* polysaccharides (PLPS) by using ATPE and evaluated its antioxidant activity. It was found that the optimal extraction conditions are as follows: Extraction time of 30 min, extraction temperature of 21.2°C, and 68.9% K_2_HPO_4_-20%[Chol]Cl, and the extraction ratio of 68.53% ± 0.29% under the above conditions. PLPS with the weight of 15.2 kDa was consisted of Ara (1.0%), Xyl (1.5%), Man (3.4%), Glc (25.2%), and Gal (1.1%). Moreover, PLPS could effectively scavenge free radicals and show good antioxidant activity (36). Hu et al. extracted *Lycium barbarum* L. fruits polysaccharides (LBPs) by using ATPE and optimized the extraction process. The results show that optimal extraction conditions are as follows: Solid-to-liquid ratio of 1:30 g/mL, extraction time of 4 min, and tissue-smashing power of 8,000 rpm, and the yield of LBPs was 24.79 mg/g under the above extraction conditions. In addition, compared with HWE and UAE, the extraction rate of ATPE was the highest (37). Mao et al. extracted and separated polysaccharide from *Grifola frondosa* by using ATPE and anion exchange chromatography, respectively. It was found that the optimized extraction conditions were as follows: pH of 3.22, ethanol volume fraction of 27.9%, and ammonium sulfate mass fraction of 17%. A homogeneous polysaccharide fraction (CGFP) with the weight of 2,954 kDa was obtained, and it was mainly consisted of Glu, and CGFP was a rod-shaped network structure with smooth surface. Moreover, CGFP could improve the levels of SOD, CAT, GSH-Px, and reduce the levels of MDA, LDH, and ROS, suggesting that CGFP can be used as a natural antioxidant with broad development and application prospects (38). Li et al. prepared polysaccharide from *Schisandra chinensis via* ATPE and optimized the extraction conditions based on BBD-RSM. It was found that the optimal parameters were as follows: K_2_HPO_4_ addition amount of 1.0 g, PEG6000 addition amount of 1.8 g, centrifugation time of 9 min, and solvent volume of 5 mL, and the yield of polysaccharide was 0.95 mg/g under the above extraction conditions. Through the above reports, it is found that the yield of polysaccharides obtained by ATPE is significantly better than that of traditional HWE (39). However, how to quickly find the optimal ATPS still needs further research.

With the advancement of extraction technology, some combined extraction methods, namely, ultrasound assisted aqueous two-phase extraction (UATPE) and ultrasound assisted enzyme extraction (UAEE), have become promising substitutes for the traditional extraction methods of TCMPs. UATPE method has the advantages of environmentally friendly, high extraction efficiency, low solvent and energy consumption (70). Thus, UATPE method is widely used to extract polysaccharides from *Cornus officinalis fruit, Ziziphus Jujuba cv., Lilium davidiivar. unicolor salisb*, and *Lilium lancifolium* thunb (40–43) (Table 1). UAEE, as a combination of UAE and EAE extraction methods, can be a good method to solve the disadvantages of traditional extraction methods. UAEE has the some merits, such as mild reaction conditions, lower investment costs and energy requirements, simplified manipulation, and environmental protection. Currently, UAEE method is used to extract polysaccharides from *Lonicerae japonica* leaves, *Momordica charabtia* L, *Cucurbita moschata*, corn silk, *Armillaria mellea*, and *Hylocereus undatus* to achieve the maximum yield of polysaccharides (44–49). The combined extraction method can improve the yield of polysaccharides, shorten extraction time, and reduce extraction temperature. However, there are still many problems in the combined extraction method, such as difficult to control the extraction process and complex influencing factors. The further research on extraction methods is needed to efficiently extract polysaccharides from TCM and maintain their biological activities, and establishing a database containing different types of polysaccharides extraction schemes of specific TCM is the key to ensure the utilization of polysaccharides to a large extent.

## Isolation and purification of TCMPs

Due to the limitations of extraction methods, the crude polysaccharides contain various impurities, such as proteins, fat, polypeptides, oligosaccharides, and ash (71). These substances will affect the biological activities, stability, and final product quality of polysaccharides. Moreover, the various impurities make it difficult to evaluate the structure-activity relationship of polysaccharides. Therefore, it is necessary to further isolate and purify the crude TCMPs. Figure 1 displays the different TCMPs purification methods. Currently, trichloroacetic acid (TCA) and Sevage methods are mainly used to remove proteins in crude TCMPs. Sevage method is based on the principle that proteins can be denatured in some organic solvents. This method has many advantages, such as mild conditions, simple operation, high purification efficiency reducing the impact on the structure and activity of TCMPs (72). However, this method has disadvantages of high purification cost and long time-consuming (73). TCA method is based on denaturation of proteins by TCA solution. Denatured proteins can be removed by centrifugation, but this method easily leads to the TCMPs degradation (74). Notably, column chromatography, ion exchange chromatography, and gel filtration chromatography are widely used in the purification of polysaccharides. Zhu et al. purified the crude turmeric polysaccharides (TPs) by anion-exchange chromatography to obtain four polysaccharide fractions (TPs-0, TPs-1, TPs-2, and TPs-3). It was found that TPs-0 and TPs-1 were composed of Glu, Gal, and Ara, whereas TPs-2 and TPs-3 were primarily consisted of Rha, Glu, Gal, Ara, Xyl, galacturonic acid, and glucuronic acid. Additionally, compared with TPs-0, TPs-1, TPs-3, and TPs, TPs-2 showed the strongest antioxidant activity (15). Chen et al. isolated and purified the polysaccharides from *Taraxacum officinale* by DEAE-52 and Sephadex G-100 to get high purified polysaccharides fraction (TOP) with the weight of 1.7 kDa. The results show that TOP had a backbone composed of (1 → 4)-β-D-Glcp with branching at O-2 of (1 → 2,4)-β-D-Glcp. Moreover, TOP could effectively scavenge DPPH radicals and inhibit the HepG2 cells proliferation, suggesting that TOP can be used as a new natural antioxidant and anti-tumor drug for disease prevention and treatment (24). Xu et al. purified the crude *Eucommia ulmoides Oliver* leaf. polysaccharides (EUOLPs) by anion exchange chromatography. It was found that a high molecular weight and polydispersity polysaccharides was obtained, and purified polysaccharides belonged to β-type acidic heteropolysaccharide structure with glucan group and high degree of branching (28). Tan et al. isolated and purified the crude *Cornus officinalis* polysaccharides (COPs) by using DEAE-52 and Sephadex G-100 chromatography. The results display that a homogenous fraction (COPs-4-SG) with the weight of 33.64 kDa was obtained and consisted of galacturonic acid, Ara, Man, Glu, and Gal with the molar ratio of 34.82:14.19:6.75:13.48:12.26 (40). Wu, Huang, and Xiang purified the polysaccharides from *Lonicerae japonica* leaves (LJLP) *via* DEAE-52 and Sephadex G-100 and evaluated its antioxidant activity. It was found that LJLP was mainly comprised of Gal (32.3%), Glu (20.9%), and Rib (15.2%). In addition, LJLP could improve SOD, GSH-Px, CAT activities, whereas LJLP could decrease the levels of MDA in both serum and liver (44). Arab et al. used the DEAE-52 cellulose column to purify the crude polysaccharides from *Ocimum album* L. seed for separating various acidic polysaccharides and neutral polysaccharides. The results show that two fractions, namely, OAP-1A was obtained and its average molecular weight was 593 kDa. In addition, OAP-1A showed strong scavenging activity against DPPH radical (50). Analysis of the above literature found that the purified polysaccharides fractions need to be dialysis and freeze-drying. Finally, the contents of polysaccharides and proteins in the purified fractions are determined by the phenol-sulfuric method and Bradford's method, respectively. Table 1 shows the different extraction and purification methods of TCMPs.

## Structure characteristics of TCMPs

The research shows that the structure of TCMPs is complex and diverse. It is necessary to analyze theirs structural characteristics in detail to better understand TCMPs. Currently, the structural analysis of TCMPs has mainly focused on the determination of molecular weight (M_W_), monosaccharide composition (MC), glycosidic bond type, glycosidic bond position, monosaccharide sequence, and spatial conformation. Furthermore, the analytical methods for the chemical characterization of TCMPs mainly include gas chromatography (GC), high performance liquid chromatography (HPLC), nuclear magnetic resonance (NMR), methylation analysis, Fourier transform infrared spectroscopy (FT–IR), gas chromatography-mass spectrometry (GC–MS), and Smith degradation analysis (75). Table 1 summarizes the existing information on the basic structural characteristics of TCMPs.

### Monosaccharide composition

Notably, polysaccharides are composed of various monosaccharides connected by glycosidic bonds. Firstly, the monosaccharide composition of TCMPs is determined by acid hydrolysis to decompose their glycosidic bonds. After hydrolysis, some polysaccharides need to be neutralized, filtered, derivatized, and then passed through some analytical chemical instruments, including GC, HPLC, PC, TLC, and HPCE, etc. Nowadays, the most common monosaccharide components of TCMPs are glucose (Glu), fructose (Fru), fucose (Fuc), mannose (Man), arabinose (Ara), rhamnose (Rha), galactose (Gal), and xylose (Xyl) (76). Lu et al. prepared the polysaccharide from *Laminaria japonica* (LJPA) by using citric acid extraction. The results show that LJPA with the weight of 17.12 kDa was consisted of Rha (4.51%), Fuc (20.27%), Xyl (12.43%), Man (12.81%), Glu (10.29%), and Gal (39.69%). Moreover, LJPA showed high antioxidant capacities including ORAC, ABTS^+^ radical scavenging activity and reducing power (16). Xu et al. prepared polysaccharides from *Eucommia ulmoides* oliver leaf and purified crude polysaccharides by anion exchange chromatography. The results show that a homogeneous fraction (EULP) was obtained and composed of Rha (7%), Ara (4%), Gal (6%), Glu (14%), Xyl (1%), Man (2%), Glucuronic acid (3%), and Galacturonic acid (1%) (28). Meng et al. prepared the crude polysaccharides from *Salvia miltiorrhiza* (SMPs) and then purified SMPs by DEAE Sepharose Fast Flow and Sephadex G-100 chromatography. It was found that a novel polysaccharide termed SMP1 was obtained and comprised of Glu, Gal, and Fru in a molar ratio of 1:1.67:1.12, and the molecular weight of SMP1 was 6.087 kDa (29). Tan et al. prepared polysaccharides from *Cornus officinalis* fruit (COPs) by UATPE and purified the crude COPs by DEAE-52 and Sephadex G-100 chromatography in turn. It was observed that COPs-4-SG with molecular weight of 33.64 kDa was obtained and composed of Gal acid (34.82%), Ara (14.19%), Man (6.75%), Glu (13.48%), and Gal (12.26%) (40).

### Molecular weight

The average M_W_ of TCMPs is the critical parameter to determine its chemical properties, and theirs biological activities are closely related to the average M_W_ of TCMPs. Currently, the M_W_ of TCMPs is usually determined by HPLC, HPLC–ELSD, high performance gel permeation chromatography (HPGPC), light scattering, gel permeation chromatography (GPC), size exclusion chromatography, and viscosity methods (77). In particular, HPGPC, as a combination of GPC and HPLC, is usually used for polysaccharides analysis and provides molar mass distribution of polysaccharides. Compared with GPC, HPGPC has the merits of accuracy, good reproducibility, high sensitivity, short analysis time, simple operation, and reliable results. However, this method has high requirements for instruments. Mao et al. extracted polysaccharide from *Grifola frondosa* by HWE and then purified the crude polysaccharide *via* DEAE-52 and Sephadex G-100 to produce the purified polysaccharide (CGFP). The results show that CGFP was mainly consisted of Glu, and the M_W_ of CGFP was 2,954 kDa (38). Zhang et al. prepared the homogenous polysaccharides fraction (AGP) from *Glycyrrhiza* inflata by different purification methods and evaluated its α-glucosidase inhibitory activity. It was found that AGP with molecular weight of 2.89 kDa was composed of Rha (1%), Ara (2.33%), Xyl (2.85%), Man (0.69%), Glc (3.05%), and Gal (1.54%). Moreover, AGP showed significant inhibition α-glucosidase activity (51). Chen et al. extracted and purified the corn silk polysaccharides (CSPS) by UAEE and anion exchange chromatography, respectively. It was found that CSPS with the weight of 105.2 kDa was comprised of Rha, Ara, Xyl, Man, Gal, and Glu with molecular ratios of 4.17:17.33:5.59:18.65:19.11:35.14 (47). Mutaillifu et al. separated and purified the *Glycyrrhiza* glabra polysaccharides (GPN) by using DEAE-cellulose 52 column and Sephadex G-100 column. The results show that the molecular weight of GPN was 38.7 kDa, and GPN was composed of Glc, Man, Ara, and Gal (78). Lu et al. and Kong, Yao, & Zhang, extracted and purified the polysaccharides from *Laminaria japonicaby* and *Ganoderma lucidum*, respectively. It was found that the molecular weights of LGPS and ASP were 17.12 and 80.00 kDa, respectively (16, 17). It is worth noting that the great difference in molecular weight may be largely due to different materials and experimental conditions.

### Chemical structure

The biological activities of TCMPs are closely related to theirs structure. Increasing researchers began to investigate the position and configuration of glycosidic bond, the type of ring structure, the position and proportion. However, the literature on the chemical structure of TCMPs is still relatively insufficient due to the limitations of identification methods. Table 1 shows the structural information of TCMPs reported in the current literature. At present, the main methods (NMR, GC–MS, FT–IR, and Smith degradation) are determined the structural characteristics of TCMPs. Chen et al. extracted polysaccharides from *Taraxacum officinale* by using UAE and purified the crude polysaccharide to produce a novel neutral polysaccharide (TOP), and then identified its structure *via* methylation and NMR analysis. It was found that the molecular weight of TOP was 1.7 kDa, and TOP had a backbone composed of (1 → 4)-β-D-Glcp with branching at O-2 of (1 → 2,4)-β-D-Glcp. Moreover, TOP showed good antioxidant and antitumor activities (24). Yi et al. prepared polysaccharide from longan pulp by HWE, purified the crude polysaccharide *via* ion-exchange chromatography and gel filtration chromatography, and then identified its structure by using NMR analysis. The results show that a homogeneous polysaccharide fraction (LPIIa) with molecular weight of 44.7 kDa was obtained and mainly composed of (→ 6)-Glc-(1 →, → 5)-Ara-(1 →, → 4)-Man-(1 → and → 6)-Gal-(1 →). Additionally, LPIIa showed immunomodulatory activity by stimulating macrophages (19). Bao et al. extracted the crude polysaccharides from longan fruit pericarp by HWE, isolated the crude polysaccharides through Sephadex G-100 gel filtration column to get a homogeneous polysaccharides fraction (PLFP), and then determined its structure *via* methylation analysis, GC–MS, and NMR. It was found that PLFP with molecular weight of 420 kDa was mainly consisted of Ara (32.8%), Glu (17.6%), Gal (33.7%), and galacturonic acid (15.9%), and the backbone consisted of (→ 5)-l-Araf-(1 →, → 6)-d-Glcp-(1 →, → 3)-d-Galp-(1 →, → 3)-d-GalpA-(1 → and → 6)-d-Galp (1 →). Moreover, PLFP showed good ability to inhibit glycosylation reaction *in vitro* (18). Zhu et al. extracted and purified polysaccharide from by HWE and anion-exchange chromatography, respectively. The results show that four polysaccharide fractions (TPs-0, TPs-1, TPs-2, and TPs-3) with different molecular weights were obtained. TPs-0 was further studied by methylation, GC-MS, and NMR. The results find that TPs-0 contained a main chain of α-Araf- (1 → 4)-α-Glcp-(1 → 3)-α-Arap-(1 → 3)-β-Galp-(1 → 3,6)-α-Galp-(1 → 5)-α-Araf-(1 → 3)-β-Galp-(1 → R) (15). Furthermore, Peng et al. and Peng et al. purified the crude polysaccharides from *Laminaria japonica* to obtain the two homogenous fractions (LJP12 and WPS-2-1) and identified their structures. It was found that LJP12 with molecular weight of 2.31 kDa was composed of Ara (1.0%), Xyl (0.17%), Man (1.54%), Glc (2.64%), and Gal (0.18%). LJP12 was suggested to be 1,4-linked and 1,3,6-linked while glucose was linked by 1,6-glycosidic bond. Additionally, WPS-2-1 with molecular weight of 80 kDa was composed of Man, Rha, and Fuc in a molar ratio of 1.0:2.3:1.2, and WPS-2-1 had a backbone of array by (1 → 4)-glycosidic linkages (52, 53). Notably, the chemical structures of TCMPs products differ greatly depending on various raw materials and preparation methods. Table 1 summarizes the existing literature on the chemical structures of TCMPs.

### Chemical modification of TCMPs

Chemical modification is to modify the polysaccharides structure by chemical, physical, and biological methods. Structural modification can change the molecular structure, molecular weight, viscosity, type, position and number of polysaccharides substituents to enhance biological activities and even obtain new bioactive polysaccharides derivatives (79). Currently, the chemical modification methods include sulfation, carboxymethylation, acetylation, selenidation, and phosphorylation. Chemical modification will replace the hydroxyl group of the original polysaccharides with target groups such as sulfuric acid group, carboxymethyl group, acetyl group, selenium ester group or phosphoric acid group, and the monosaccharide composition, molecular weight, and morphological characteristics change by the above chemical modification methods.

### Sulfation modification

**Figure 2:**
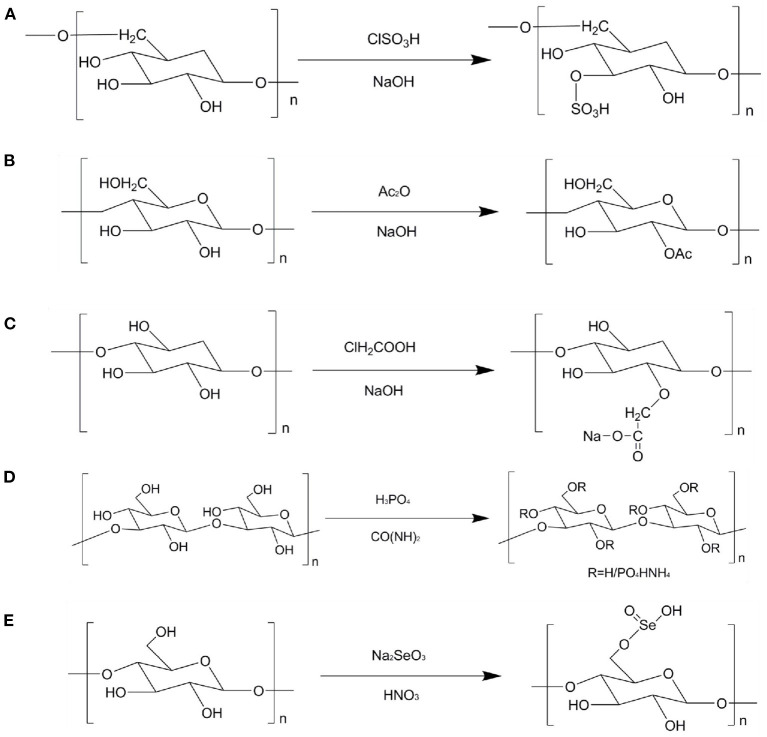
Schematic diagram of different modification of TCMPs. **(A)** Sulfation modification; **(B)** acetylation modification; **(C)** carboxymethylation modification; **(D)** phosphorylation modification; **(E)** selenium modification.

The principle of sulfation modification is to dissolve the polysaccharides and corresponding sulfation reagent in a certain solvent and react with each other under certain conditions to introduce the sulfate group into some hydroxyl groups on the residues of the original polysaccharides (80). Sulfation modification is one of the most commonly used method to modify the chemical structure of polysaccharides. This method can change the water solubility and biological activities of polysaccharides (79). Figure 2A describes the sulfation modification of polysaccharides. The common modification methods are chlorosulfonic acid pyridine, concentrated sulfuric acid, and sulfur trioxide pyridine methods. Among them, chlorosulfonic acid pyridine method has the advantages of high yield, high degree of substitution, and convenient recovery. Thus, it is the most common method to prepare sulfated polysaccharides. Geng et al. prepared the sulfated polysaccharide (SPPM60-D) by using chlorosulfonic acid pyridine method and evaluated its immunologic activity. The results show that SPPM60-D showed stronger immunologic activity than unmodified polysaccharides. In addition, SPPM60-D could activate TLR4-PI3K-IP3K signal pathway to regulate immune activity (81). Wang et al. investigated three sulfated ginger polysaccharides, namely, SGP, SGP1, and SGP2, and evaluated theirs anticoagulant bioactivity. It was found that SGP and SGP1 have triple helical structure, whereas SGP2 existed random coils. SGP, SGP1, and SGP2 displayed rough surfaces with a large number of small holes. Moreover, GP2, SGP2, and SGP could inhibit the intrinsic pathway of coagulation. Therefore, ginger polysaccharides can be used as an anticoagulant and therapeutic agent for thrombosis (82). Huang et al. prepared a sulfated polysaccharides from Chinese yam (S-CYP) by chlorosulfonic acid pyridine method and evaluated its immunological activity. It was found that S-CYP showed stronger immunological activity than CYP (unmodified polysaccharide). Additionally, S-CYP could up-regulate the levels of IgG and IgM secretion in serum, down-regulate the CD^4+^/CD^8+^ ratio, indicating that sulfated polysaccharides can improve immunomodulatory activity to some extent (83). Furthermore, the origin polysaccharides from *Laminaria japonica* and *Laminaria angustata* sulfated ginger polysaccharides were sulfated to obtain corresponding sulfated polysaccharides and found that the biological activities of sulfated polysaccharide were significantly higher than those of original polysaccharides (84, 85).

### Acetylation modification

Acetylation modification has become one of the commonly used molecular modification methods in the chemical structure modification of polysaccharides because of its fast reaction speed, mild reaction, and high product conversion rate. Acetylation modification changes the physical and chemical properties of polysaccharides by changing the orientation and horizontal order of polysaccharides. Acetylated polysaccharides will lead to changes in the extension of molecular structure, exposing the hydroxyl groups in their molecular structure, increasing the solubility of polysaccharides, thus affecting the biological activities of polysaccharides (86). Figure 2B shows the acetylation modification of polysaccharides. The acetic anhydride pyridine method uses formamide, methanol, and dimethyl sulfoxide as reaction solvents. Acetic anhydride and acetic acid are acetylating reagents. N-bromosuccinimide (NBS), 4-dimethylaminopyridine (DMAP), and pyridine are used as catalysts. Gu et al. prepared the acetylated polysaccharides derivative from *Ophiopogon japonicus* (ROH05) and evaluated its anti-pancreatic cancer activity. It was found that the molecular weight of ROH05 was 16.7 kDa, and it was composed of 1, 4-linked β-D-Gal and 1, 4, 6-linked β-D-Gal. Furthermore, ROH05A could inhibit BxPC-3 and PANC-1 cells growth in a dose-dependent manner (87). Liu et al. modified polysaccharides from *Polygonatum cyrtonema* Hua by different chemical modification methods (sulfated, acetylated, and phosphorylated). The sulfated derivative (S-PD), phorphorylated derivative (P-PD), and acetylated derivative (Ac-PD) showed good antiherpetic activity. The results suggest that the types of functional groups are very important for the anti-herpetic activity of polysaccharides. Song et al. prepared the acetylated *Pleurotus geesteranus* polysaccharides (AcPPS) and evaluated its anti-inflammatory, antioxidant, and lung protection. AcPPS could decrease the levels of lung index, TNF-α, IL-1β, IL-6, p-IκBα, ROS, MDA, TC, TG, LDL-C, HDL-C, and VLDL-C, whereas AcPPS could increase the levels of SOD, GSH-Px, CAT, and T-AOC. These results imply that the acetylated polysaccharides may be suitable as a natural food to prevent or alleviate liver injury (88). Chen and Huang prepared bitter gourd polysaccharide (P) by HWE and modified its structure *via* carboxymethylated and acetylated modifications to obtain two fractions, namely, CM-P and AcP, and evaluated theirs antioxidant activity. The results display that compared with the original polysaccharides, CM-P and AcP showed stronger free radicals scavenging ability and anti-lipid peroxidation capacity, indicating that different chemical modifications can enhance the antioxidant activity of polysaccharides from bitter gourd (89).

### Carboxymethylation modification

Carboxymethylation modification has the advantages of easy availability of reagents, low cost, and low toxicity or non-toxic substances generated by the reaction. Thus, carboxymethylation modification has become one of the commonly used methods for the chemical structure modification of polysaccharides (79, 89). Relevant studies have shown that some polysaccharides have good antitumor activity (90). However, the wide application of polysaccharides is limited due to its high molecular weight and poor solubility. Carboxymethylation modification of polysaccharides structure can effectively improve the water solubility of polysaccharides and play an important role in the biological activities of polysaccharidse. The principle of carboxymethylation modification is to react polysaccharides with monochloroacetic acid under alkaline conditions, which makes carboxymethyl introduced to some hydroxyl groups on the residues of the original polysaccharides (91). Figure 2C shows the carboxymethylation modification of polysaccharides. Ren et al. extracted the crude polysaccharides from *Cordyceps militaris* and then modified the crude polysaccharides to obtain carboxymethylated derivatives (CM-CPS) and acetylated derivatives (AC-CPS). The results imply that the carboxymethylation substitution of polysaccharide might be C-6, C-2, and the acetylation modification might be C-3, C-6 inferred from NMR analysis. In addition, CM-CPS and AC-CPS exhibited good α-glucosidase inhibitory activity. Hence, the change structures of polysaccharides have a certain impact on the biological activities. The degree of substitution and the position of substituent are the factors that affect the biological activities of polysaccharides (92). Zhao et al. prepared carboxymethylated *Schisandra* polysaccharides (CSP), purified CSP by using DEAE-52 column to obtain the main fraction (CSPP), and then investigated its structural feature and biological availability. It was found that CSPP with the molecular weight of 1.698 × 10^4^ g/mol was comprised of Man, Glu, and Gal at ratio of 1: 44.8: 3.7. The water solubility of CSPP was significantly higher than that of unmodified polysaccharides. Additionally, CSPP showed higher immunomodulating activity than unmodified polysaccharide, indicating that CSPP can be used as an effective regulatory immunosuppressant (93). Liu et al. extracted and isolated *Craterellus cornucopioides* polysaccharides and then then modified polysaccharides by carboxymethylation modification to produce two fractions (CCPs-1 and CCPs-2). The results show that CCPs-1 and CCPs-2 were consisted of Rha, Fuc, Ara, Xyl, Man, Glu, and Gal with different molar ratios. CCPs-1 and CCPs-2 were primarily connected by Man with (1 → 3)-linked. In addition, CCPs-1 and CCPs-2 showed good antioxidant activity (94). Wang, Zhang, and Zhao prepared a water-insoluble crude polysaccharides from *Tremella fuciformis* (ATP) and modified ATP structure. It was found that the degree of substitution (DS) of the four carboxymethylated derivatives varied with the molar number of chloroacetic acid. With the increase of DS content, their water solubility and biological activities increased, suggesting that carboxymethylation modification can effectively improve the potential biological properties of polysaccharides (95).

### Phosphorylation modification

Phosphorylation modification is one of the common methods of modifying the chemical structure of polysaccharides. Like other chemical modifications, it is also a covalent modification of molecular branches. After phosphorylation modification, the biological activities of polysaccharides significantly improved. Therefore, phosphorylation modification has attracted the attention of relevant fields. The principle of phosphorylation modification is that the polysaccharides react with the phosphorylation reagent to introduce the phosphate group to some hydroxyl groups on the polysaccharides residues (96). Figure 2D shows the phosphorylation modification of polysaccharides. Deng et al. obtained the water-soluble phosphorylated polysaccharides (P-DIP) by using phosphorylation modification and investigated it's the physiochemical and biological properties. It was found that the water solubility of P-DIP was significantly higher than that of DIP. P-DIP could significantly inhibit the growth of MCF-7 and B16 tumor cells in a dose-dependent manner. Moreover, P-DIP could effectively remove DPPH and OH radicals, implying that phosphorylation modification can help to improve the water solubility of natural DIP and enhance its antioxidant and antitumor activities (97). Ming et al. phosphorylation modified polysaccharides from *Chrysanthemum indicum* to obtain a single fraction (pCIPS). The results show that pCIPS was observed the polysaccharides surface features. Additionally, the anti-DHAV activity of pCIPS enhanced after phosphorylation modification (98). Feng et al. prepared the nine phosphorylated *Radix Cyathulae officinalis* Kuan polysaccharides (pRCPS) and evaluated its antiviral activity. The results observe that the pRCPS1-4, pRCPS7, and pRCPS9 showed significant anti-viral activity. In addition, phosphorylation modification could be used as a method to improve the antiviral activity of polysaccharides (99).

### Selenide modification

Selenium is an essential element of human life activities, which can enhance the antioxidant capacity of the body and play an important role in anti-cancer. Relevant experiments have confirmed that selenium polysaccharides formed by organically combining selenium and polysaccharides have a variety of biological activities, which can antagonize heavy metal poisoning and reactive oxygen species damage by improving the activity of related enzymes, blocking the DNA synthesis of cancer cells and inhibiting the growth of cancer cells (100). Selenium polysaccharides take into account the activities of selenium and polysaccharides, and selenium polysaccharides are more easily absorbed and utilized by the body. Due to its low toxicity, low side effects and easy absorption, it has attracted the attention of scientific and technological researchers. Figure 2E shows the selenium modification of polysaccharides. Sun et al. obtained the Se-containing polysaccharide-protein complex (Se-PPC) and evaluated its anti-tumor activity. It was found that Se-PPC could prominently inhibit the growth of cancer cells though induction of apoptosis, and increase the population of apoptotic sub-G1 phase cells, up-regulate the expression of anti-apoptotic (Bcl-2 and Bcl-XL), indicating that Se-PPC is a promising new organic selenium compound, which has the potential to treat human cancer (101). Lian et al. prepared polysaccharides from *Glycyrrhiza uralensis* (GUP) and modified GUP structure *via* selenide modification to produce the selenized GUP (SeGUP) and evaluated its antioxidant activity. The thermal stability and particle size of SeGUP were significantly different from those of GUP. Moreover, SeGUP showed greater antioxidant activities *in vitro* and *in vivo* when compared to GUP. These results show that selenide modification can significantly enhance the antioxidant activity of SeGUP, and SeGUP has the potential to be developed as a natural antioxidant (102). Furthermore, the biological activities of polysaccharides from *Codonopsis pilosula* and *Ganoderma lucidum* mycelia could be significantly improved by selenium modification (103, 104).

The research on the structural modification of polysaccharides has made great achievements. At present, a variety of polysaccharides modification methods have been mastered. However, various modification methods still have different shortcomings. Although the chemical modification method is highly targeted, it can change the structural form and state of a certain part to obtain a certain biological activities of the target polysaccharides. Nevertheless, it is difficult to treat the organic reagent, which is easy to cause environmental pollution. In addition, the reaction process is not easy to control, which is easy to introduce impurities and cause side effects of polysaccharides. The physical modification method is easy to operate and is not easy to damage the structure of polysaccharides. However, this method has no effect on the inherent biological activities. Therefore, under the guidance of science and technology, we should try to combine various methods to modify the structure of polysaccharides in the future. In addition, we should strengthen the research on the structure-activity relationship of modified polysaccharides, and clarify the correlation between structural characterization and biological activities.

## Biological activities of TCMPs

Growing studies have indicated that polysaccharides, as one of the key bioactive components of TCM, have various biological activities, such as antioxidant, anti-aging, immunomodulatory, hypoglycemic, hypolipidemic, anti-tumor, anti-inflammatory, and other activities. Currently, increasing TCMPs are used to treat various chronic diseases or developed into special health products to meet the needs of specific populations. Figure 1 summarizes the biological activities of TCMPs.

### Antioxidant and anti-aging activities

**Figure 3:**
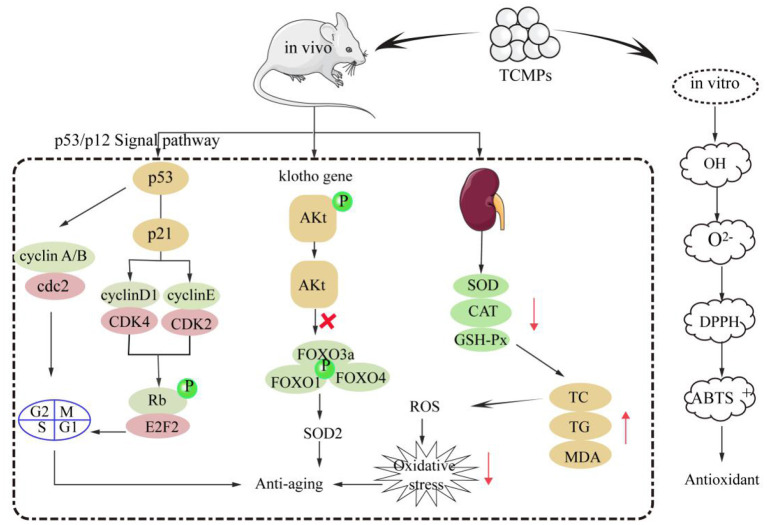
The main antioxidant and anti-aging mechanisms of TCMPs.

Oxidative stress will lead to the damage of lipids, proteins, DNA, and lipid membranes in the body, and eventually induce aging (105). Growing studies have shown that TCMPs have strong antioxidant and anti-aging effects (8, 106). TCMPs mainly show their antioxidant effect *in vitro* by scavenging free radicals. In addition, TCMPs can play an anti-aging role by activating various antioxidant enzymes, regulating the expression of anti-aging gene klotho and p53/p12 signaling pathway. TCMPs can improve theirs antioxidant activities *in vitro* by scavenging OH, O^2−^, DPPH, and ABTS^+^ radicals, and the scavenging effect of TCMPs is basically equivalent to that of V_C_ (9, 107). The mechanism of TCMPs scavenging free radicals might be that the OH group in TCMPs can provide hydrogen and combine with OH radical to promote the scavenging radical. Moreover, there may be electrophilic groups (aldehydes or ketones) in TCMPs, which will release hydrogen atoms and scavenge O^2−^ radical. The scavenging of DPPH and ABTS^+^ radicals may be related to the monosaccharide composition, glycosidic bond and configuration of TCMPs (3, 108, 109). TCMPs could significantly increase the activities of various enzymes such as SOD, CAT, and GSH-Px in the body by up regulating the expression levels of related antioxidant genes. Moreover, TCMPs could significantly reduce the levels of MDA, TC, and TG, thereby reducing the number of intracellular free radicals, improving the ability of the body to resist oxidative stress, reducing oxidative damage, and delaying aging (110, 111). Furthermore, TCMPs could up regulate the expression of anti-aging klotho gene in liver and kidney to delay aging in mice. The klotho protein started the intracellular signal transduction process by binding to specific receptors on the surface of cell membrane. This signal transmission could inhibit the phosphorylation process of specific target enzymes (PI3K and PKB), resulting in the dephosphorylation of Akt, and Akt dephosphorylation could inhibit the phosphorylation of forkhead transcription factors FOXO family (FOXO1, FOXO3a, FOXO4, etc.), and then directly regulate and promote the expression of SOD2 to eliminate intracellular reactive oxygen species, reduce oxidative stress, and delay the aging of the body (112). Additionally, p53/p12 signaling pathway plays an important role in the regulation of cell aging induced by oxidative stress. p53 and p12 proteins are the key proteins in the regulation pathway of cell aging. When oxidative stress occurred, p53 protein was activated and its expression levels increased, which activated p21 protein and significantly increased its expression levels, eventually leading to the occurrence of aging (113). The mRNA expression levels of p16 and p21 in aging rats induced by D-galactose decreased after gavage *Astragalus* polysaccharides. Western blot analysis shows that *Astragalus* polysaccharides could significantly down regulate the proteins expression levels of p53 and p21 in rat liver and brain, thereby down regulating the expression levels of aging genes (114). Therefore, it is inferred that the main antioxidant and anti-aging mechanisms of TCMPs is shown in Figure 3.

### Immunomodulatory activity

**Figure 4:**
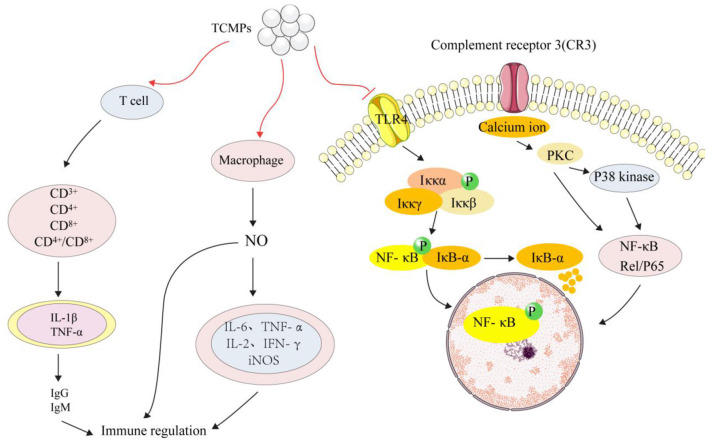
The main immunomodulatory mechanism of TCMPs.

Immunomodulatory activity is considered to be an important ability to improve the physical defense mechanism of the elderly and cancer patients. A large number of studies have shown that TCMPs have the effect of immune regulation (115, 116). Hao and Zhao prepared the water-soluble yam polysaccharides (WYPs) by HWE and evaluated its immunomodulatory activity. It was found that WYPs (500 mg/kg) could increase spleen and thymus indices by 22–42%, enhance macrophages' phagocytosis and NK cell activity. Moreover, WYPs (500 mg/kg) could elevate the levels of IL-2 and IFN-γ in the splenocytes, and IL-1β, IL-6, TNF-α, and iNOS in the splenocytes. Furthermore, WYPs could increase the levels of IgM, IgA, and IgG in serum, suggesting that WYPs can be developed into potential immunosuppressants (117). Carboxymethyl *Poria cocos* polysaccharides activated macrophage centered innate immunity and increased the expression levels of activation mmacrophage membrane receptors (CD^3+^, CD^4+^, CD^8+^, and p38 kinases) in RAW264.7 macrophages (118). Additionally, *Poria cocos* polysaccharides could restore CD4^+^/CD^8+^ in immune injured mice, activate T cells, release a large amount of tumor necrosis factor (TNF-α and IL-1β), stimulate the production of IgG and IgM in serum, activate the immune system and enhance immune regulation function (119). NF-κB is a transcription factor that regulates various genes related to immune and inflammatory responses. In the cytoplasm of unstimulated cells, NF-κB binds to NF-κB inhibitor (IκB) and becomes an inactive complex form. IκB kinase (IKK) complex was activated when cells were stimulated. IKK family catalyzed IκB-α phosphorylation and dissociation from NF-κB. IκB-α degraded and induced nuclear heterotopia of NF-κB, transforming NF-κB into an activated form (120). Increasing studies have shown that TCMPs could activate RAW 264.7 macrophages by activating NF-κB signaling pathway, release a large amount of NO, promote the expression levels of IL-6 and TNF-α, which ultimately played its role in immune regulation. Figure 4 shows the main mechanism of TCMPs in immune regulation.

### Hypoglycemic and hypolipidemic activities

**Figure 5:**
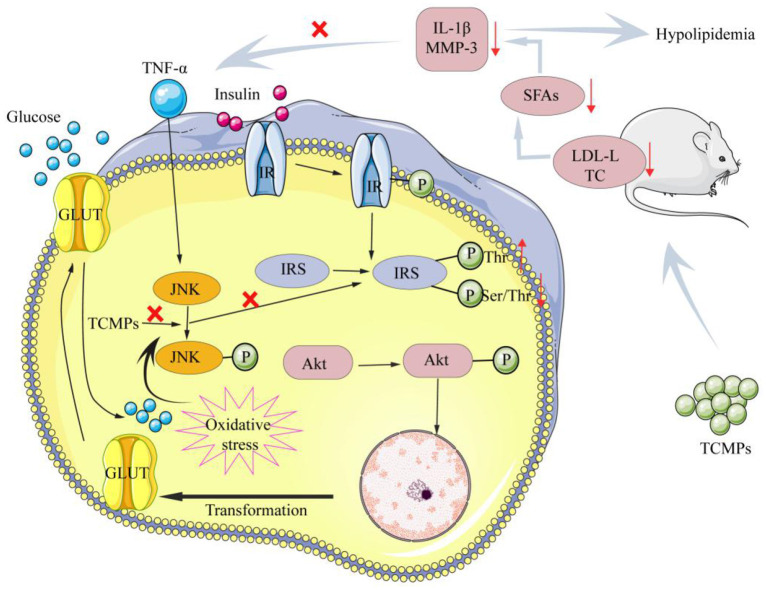
The main hypoglycemic and hypolipidemic mechanisms of TCMPs.

At present, the number of “three highs” in China is gradually increasing. In addition, the “three highs” have an obvious trend of being younger, younger, and grassroots (121). Diabetes is a chronic endocrine metabolic disease characterized by long-term hyperglycemia, which is one of the three major diseases in the world (122). Hyperlipidemia is a common and frequently occurring disease in clinic, which is characterized by high levels of cholesterol (TC), triglycerides (TG), low-density lipoprotein cholesterol (LDL-C) and low levels of high-density lipoprotein cholesterol (HDL-C) (123). Currently, some drugs used to treat hypoglycemia and hypolipidemia in clinic have certain side effects. Therefore, it is very important to find green and natural drugs to reduce blood glucose and blood lipids. Qian et al. extracted the *Trichosanthes* peel polysaccharide (TPP), and then purified TPP by using Sephadex G-100 column to produce the homogeneous component (TPP-1), and evaluated its hypoglycemic activity. It was found that TPP-1 was consisted of Ara, Man, Glc, and Gal with a molar ratio of 1.00:3.27:4.26:6.01. Moreover, TPP-1 could reduce the blood glucose levels in hyperglycemia mice. TPP-1 could increase significantly the contents of insulin and total superoxide dismutase of the hyperglycemia mice, whereas TPP-1 could decrease the levels of biochemical indexes, including MDA, creatinine, triglyceride, LDL-C, and blood urea nitrogen (124). Cao et al. isolated and purified polysaccharide from *Sargassum pallidum* to obtain the homogeneous component (PSP-1) with the molecular weight of 1,036 kDa, and determine its antioxidant and hypoglycemic activities. The results show that PSP-1 was comprised of Fuc (18.45%), Ara (2.15%), Gal (19.06%), Glu (1.89%), Xyl (16.07%), Man (1.00%), Galacturonic acid (5.74%), and Glucuronic acid (20.09%). PSP-1 showed good DPPH and OH radicals scavenging activities. Additionally, PSP-1 could remarkably inhibit α-amylase and α-glucosidase activities, improve glucose consumption, glycogen synthesis and the activities of pyruvate kinase and hexokinase in insulin-resistance HepG2 cells, suggesting that PSP-1 can be used as a potential antioxidant and hypoglycemic agent for disease prevention and treatment (125). Lee et al. studied whether yam polysaccharides (YPs) have an effect on insulin resistance. The results show that YPs played an important role in inhibiting insulin resistance induced by TNF-α or ROS. The potential hypoglycemic mechanism of YPs was that YPs could inhibit the Tyr phosphorylation of IRS, increase the levels of IR and phosphorylated serine/p-Akt, thus increasing the activity of Akt/PKB, inhibiting the increase of glucose in mice, and reducing the levels of blood lipids in mice (126). Furthermore, yam polysaccharides could reduce the levels of LDL-C and total TC, inhibit the production of SFAs, reduce the levels of IL-1β and TNF-α in serum, and down regulate the expression levels of MMP-3 protein in visceral adipose tissue to improve insulin resistance and reduce blood lipids (127). Figure 5 shows possible hypoglycemic and hypolipidemic mechanisms of TCMPs.

### Anti-tumor activity

Although great progress has been made in the diagnosis and treatment of human malignant tumors, the long-term prognosis is still deal. At present, the treatment of cancer mostly depends on chemical drugs. However, long-term use of chemical drugs will lead to the decline of body immunity. Therefore, it is of great significance to find natural anti-tumor drugs with high efficiency, low toxicity, and low side effects. TCMPs, as natural active ingredients, are used to inhibit the growth of tumor cells through different signal pathways. Zhao et al. extracted *Lentinus edodes* polysaccharides (LEPs) using EAE, and then purified the crude LEPs to obtain two homogeneous polysaccharides fractions (LEPs-1 and LEPs-2). These results observed that LEPs-1 and LEPs-2 could markedly inhibit the proliferation of HCT-116 and HeLa cells, indicating that LEPs can be used as a potential natural anti-tumor candidate (128). Ma et al. isolated two homogeneous polysaccharides fractions (CPPS-1 and CPPS-2) *via* ultrafiltration method and determined their cytotoxicity and antioxidant activity. These results found that the antioxidant activity of CPPS-1 was significantly better than that of CPPS-2. Moreover, CPPS-1 could down-regulate the expression of Bcl-2 and up-regulate the expression of p53 and Bax, suggesting that the anti-tumor activity of CPPS-1 is closely related to its induction of apoptosis by activating the mitochondrial apoptosis pathway. It is noteworthy that the abnormal activation of JAK/STAT and ERK1/2 pathways were closely related to the proliferation, differentiation, invasion, and apoptosis of cancer cells. Reducing cancer toxicity is also one of the anti-tumor mechanisms of TCMPs. TCMPs could inhibit tumor recurrence and metastasis, and reduce organ damage caused by chemotherapy, radiotherapy and drugs in the process of cancer treatment (129). TCMPs could interfere with TGF signaling pathway and reduce the relative expression levels of TGF-βRI (transforming growth factor-beta receptor I) and metastatic proteins in gastric cancer transplanted mice. Additionally, TCMPs could reduce the phosphorylation of FAK and AKT, and significantly inhibit the metastasis and invasion of lung cancer CL1-5 cells (130). TCMPs could reduce the expression levels of MMP-9 and VEGFA proteins in HeLa cells and inhibit the formation of neovascularization, which was not conducive to the migration, invasion, and metastasis of tumor cells (131). The literature review found that TCMPs have significant antitumor activity. However, molecular mechanisms of their antitumor activity are still unclear. Hence, the following research still needs to continue to explore the molecular mechanism of TCMPs anti-tumor.

### Anti-inflammatory activity

The body can secrete immune factors to improve the immune function when pathogens invade the body. However, excessive immune response such as inflammatory response will cause damage to the body (8, 105). Inflammation is the key factor in the development of various pathological processes such as cancer and depression (132). Jujube polysaccharides have antioxidant capacity to scavenge DPPH, ABTS^+^, and OH radicals. The evaluation results of anti-inflammatory activity *in vitro* show that the jujube polysaccharides still had strong anti-inflammatory ability. It was further inferred that jujube polysaccharides could regulate the activity of intracellular antioxidant enzymes by scavenging excess free radicals to interfere with the relevant targets in the inflammatory reaction and further enhance its anti-inflammatory effect (133). Cytokines are mediators of interaction between cells and play an important role in the occurrence and maintenance of inflammation. The anti-inflammatory cytokines of TCMPs are mainly tumor necrosis factor, including IL-6, IL-1β, and IL-10. Macrophages play an important role in the development of inflammation. Liu et al. found that carboxymethyl *Poria cocos* polysaccharide acted on trinitrobenzene sulphonic acid (TNBS) induced colitis in mice, and myeloperoxidase (MPO) activity and MDA content in colon tissue decreased and protected colitis in mice by regulating potential targeted proteome and key protein metabolism. Carboxymethyl *Poria cocos* polysaccharides could interfere with the down regulation signaling pathways of NF-κB, myosin light chain kinase (MLCK), and phosphorylated myosin light chain (p-MLC) in TNF-α injured Caco-2 cells. In addition, polysaccharides could enhance the expression levels of intestinal tight junction protein and effectively prevent colitis (134). The above mechanisms could inhibit the production of pro-inflammatory factors, increase the levels of anti-inflammatory factors, reduce oxidative stress and intestinal barrier damage, and achieve the anti-colitis effect (135). In conclusion, it is a common method to observe the regulatory effects of TCMPs on inflammatory response by measuring the levels of cytokines. It is of great significance to study the changes of cytokines in inflammation and explore the anti-inflammatory mechanism at the molecular level. However, the structure-activity relationship and specific signal pathways of TCMPs still need to be further revealed and summarized.

### Other activities

Furthermore, TCMPs have liver protection, radiation protection, anticoagulation, and other activities. Liu, Liu, and Qian found that *Cynanchum auriculatum* flower polysaccharides have a significant protective effect on ethanol induced liver injury in mice by increasing the activity of liver antioxidant enzymes, reducing the ability of MDA lipid peroxidation and the expression of inflammatory factors (TNF-α, IL-6, IL-1β, and IL-10) (135). *Poria cocos* polysaccharides showed many biological activities, such as anti-oxidation, anti-aging, anti-fatigue, lowering blood sugar and lipid, regulating urinary system, and bacteriostasis (136). Li et al. found that *Sophora japonica* polysaccharide could significantly down regulate the expression levels of JNK phosphorylation and p38 MAPK protein phosphorylation in HaCaT cells exposed to outdoor ultraviolet (UVB) through MAPK pathway, which could effectively protect HaCaT keratinocytes from skin damage caused by UVB, and significantly inhibit UVB induced cytotoxicity (137). Overall, the findings indicate that TCMPs could be used as natural anti-radiation drug candidates.

## Conclusions and future prospects

Many diseases, such as malignant tumor, low immunity, gastroenteritis, alcoholic liver, fatty liver, depression, hyperglycemia, etc. induced by poor diet, work, environment and other factors, have aroused widespread concern about traditional Chinese medicines. In recent years, the various active functions of traditional Chinese medicines have been deeply explored, which has great potential in the development of food, drugs and health products. TCMPs combined with Chinese herbal medicines, anticancer drugs, and vaccines will be the research hotspot. The synergistic effect and structure-activity mechanism of TCMPs and other active ingredients deserve further exploration. Different edible parts, sources, storage, and processing methods of traditional Chinese medicines lead to certain differences in the content, structure, and biological activities of TCMPs. Strengthening the quality control of traditional Chinese medicine is the key to keep the chemical structure and biological activities of TCMPs reliable and consistent in food and pharmaceutical applications.

TCMPs have poor water solubility, low yield, difficult separation and purification, and low biological activities. The water-soluble TCMPs produced by modification can enhance the original biological activities or produce new biological activities. Hence, the structural modification of TCMPs is the main research direction of scholars at home and abroad. Currently, carboxymethyl TCMPs are used to develop adjuvant drugs for cancer treatment and put into the market. Great breakthroughs have been made in the market development of TCMPs. However, the study on the relationship between the structure and bioactivity mechanisms of TCMPs is relatively limited due to the limitations of spatial structure testing technology, which limits its development and application. Hence, the quality of TCMPs derivatives can be controlled by further standardizing the quality of TCMPs, deeply studying the various biological activities of TCMPs, and clarifying the fine structure and structure-activity relationship of TCMPs. Especially, the chemical modification degree and reliable pharmacokinetic study of TCMPs derivatives can promote the development of new polysaccharides or health food.

## Author contributions

HX and PL: formal analysis (supporting), investigation (equal), and writing-original draft (equal). JB, YG, and YS: formal analysis (supporting) and investigation (equal). JT: funding acquisition (equal), project administration (equal), and writing-review and editing (equal). All authors contributed to the article and approved the submitted version.

## Conflict of interest

The authors declare that the research was conducted in the absence of any commercial or financial relationships that could be construed as a potential conflict of interest.

## Publisher's note

All claims expressed in this article are solely those of the authors and do not necessarily represent those of their affiliated organizations, or those of the publisher, the editors and the reviewers. Any product that may be evaluated in this article, or claim that may be made by its manufacturer, is not guaranteed or endorsed by the publisher.
